# Characterizing surveillance-associated anxiety following curative-intent surgery for gastrointestinal cancer: a mixed methods approach

**DOI:** 10.1007/s11764-025-01781-w

**Published:** 2025-04-29

**Authors:** Natalie M. Bath, Rakhsha Khatri, Patrick L. Quinn, Joanne Kim, Mary Dillhoff, Aslam Ejaz, John Hays, Anne Noonan, Emily Huang, Alex Kim, Timothy M. Pawlik, Jordan M. Cloyd

**Affiliations:** 1 Department of Surgery, The Ohio State University Wexner Medical Center, Columbus, OH USA; 2 Department of Biomedical Informatics, The Ohio State University College of Medicine, Columbus, OH USA; 3 Department of Surgery, University of Illinois Hospital and Health Sciences System, Chicago, IL USA; 4 Department of Internal Medicine, The Ohio State University Wexner Medical Center, Columbus, OH USA; 5 Department of Surgery, UT Southwestern Medical Center, Dallas, TX USA; 6 Division of Surgical Oncology, The Ohio State University Wexner Medical Center, 410 W 10th Ave, N943 Doan Hall, Columbus, OH 43210 USA

**Keywords:** Cancer surveillance imaging, Scan-associated anxiety, Gastrointestinal cancers, Mixed-methods analysis

## Abstract

**Purpose:**

Imaging is often an essential component of cancer surveillance following curative-intent surgery but can be distressing for some patients. While this phenomenon has been studied among patients undergoing active cancer treatment and screening, the frequency and severity of “scanxiety” among patients undergoing surveillance after curative-intent cancer surgery is poorly understood.

**Methods:**

A cross-sectional mixed-methods analysis of patients with a history of resected gastrointestinal (GI) or hepatopancreatobiliary (HPB) cancer undergoing routine cancer surveillance was conducted. After their imaging was completed but before meeting with their provider, patients completed the Impact of Events Scale-revised (IES-r) and the Hospital Anxiety and Depression Scale (HADS) anxiety subscale surveys. IES-r and HADS-anxiety scores ≥ 24 (scale 0–88) and ≥ 11 (scale 0–22), respectively, were considered clinically meaningful. Semi-structured interviews were conducted among a convenience sample of patients, which were subsequently transcribed and then coded using an inductive approach.

**Results:**

Among 101 participants, mean age was 62 ± 13.9 years old and 52% were male. The most common diagnoses were cancers of the pancreas (23.8%), appendix (20.8%), and colon & rectum (20.8%). Mean time since surgery was 30.7 ± 28.0 months. Overall IES-r and HADS scores were 12.1 ± 14.7 and 5.2 ± 4.7, respectively. On multivariable linear regression analysis, a previous mental health diagnosis was associated with increased HADS, total IES, and all IES subscale scores whereas older age was associated with lower HADS and total IES scores (all p < 0.05). African American race, public insurance, and longer distance traveled were associated with increased IES hyperarousal scores. Qualitative analysis highlighted the psychological symptoms associated with surveillance imaging, its impact on daily living, and primary coping strategies.

**Conclusions:**

In this cross-sectional study of patients with resected GI and HPB cancers, symptoms of anxiety and post-traumatic stress around the time of routine cancer surveillance were common but relatively mild. A prior mental health diagnosis and younger age may be risk factors for greater symptomatology.

**Implications for Cancer Survivors:**

Future research should work to identify which patients are at highest risk for surveillance-associated anxiety so that patient-centered interventions can be designed and studied in this growing patient population.

**Supplementary Information:**

The online version contains supplementary material available at https://doi.org/10.1007/s11764-025-01781-w.

## Introduction

Imaging plays a critical role in the diagnosis, treatment, and follow-up of patients with cancer. Imaging results may significantly impact treatment plans and are also used to detect cancer recurrence among patients who have undergone curative intent surgery. As a result, surveillance imaging and follow-up visits can be anxiety-provoking to cancer patients. Additionally, patients commonly now have direct access to their test results prior to meeting with a provider, which may cause additional distress regarding the interpretation of these results. The term “scanxiety” has been used to describe the distress leading up to, during, and after scans have been performed [1, 2]. This surveillance-associated anxiety has been shown to lead to significant disruptions to daily life and impaired quality of life [3].

Surgical resection is a key component of curative-intent treatment for most gastrointestinal (GI) cancers. Following surgical resection, it is common to undergo repeat imaging and follow-up visits every 3 to 6 months for at least 5 years but often extending to 10 years or even lifetime. Particularly in patients who have successfully undergone surgical resection for cancer, surveillance imaging may lead to psychological and emotional distress regarding fear of cancer recurrence (FCR) [4]. Additionally, patients may experience discomfort from the scan, stress around scheduling, and/or financial toxicity as a result [5]. As a result, cancer patients may be at an increased risk for emotional distress and the development of post-traumatic stress symptoms (PTSS) around the time of their surveillance [1, 2].

Previous studies have focused on the prevalence and factors associated with surveillance-associated anxiety among patients undergoing cancer screening or active cancer treatment [6–8]. However, the experiences of patients undergoing surveillance after curative-intent treatment may uniquely differ from others undergoing cancer imaging due to the significant implications of scan results on their prognosis. Notably, a recent systematic review of existing literature found that the frequency and severity of scanxiety among patients undergoing surveillance after curative-intent surgery is poorly understood [9, 10]. Therefore, the objective of this study was to better characterize scanxiety among patients undergoing surveillance imaging after curative intent surgery using a mixed methods approach and to identify factors associated with high levels of scan-related anxiety.

## Methods

### Study design & population

This is a cross-sectional mixed-methods study evaluating patients with a history of resected gastrointestinal (GI) or hepatopancreatobiliary (HPB) cancer. Patients were enrolled after undergoing surveillance imaging but before meeting with a provider. Specifically, prior to their appointment with their provider, a trained research assistant approached them about the study, completed informed consent, and administered the research questionnaires. Patients were then immediately seen by the medical providers and their test results were reviewed. Inclusion criteria consisted of age ≥ 18 years old, history of resected GI or HPB cancer, and English speaking. Exclusion criteria included receipt of active cancer treatment or receipt of test results from the provider at the time of study participation.

### Measures

We hypothesized that following curative-intent treatment, cancer survivors’ experiences during surveillance may be influenced primarily by anxiety and PTSS. Therefore, the Hospital Anxiety and Depression Scale and the Impact of Event Scale-revised were used in the study. Hospital Anxiety and Depression Scale anxiety subscale (HADS-anxiety), a 7-item questionnaire (each item rated 0–3) is a validated measure of anxiety with scores between 8–10 and ≥ 11 (out of 21) indicating borderline abnormal or abnormal symptoms, respectively [11, 12]. Impact of Event Scale-revised (IES-r) is a 22-question survey (each item rated 0–4) designed to measure symptoms of intrusion, avoidance, and hyperarousal that may be associated with highly stressful and traumatic events. IES-r total scores ≥ 24, ≥ 33, or ≥ 37 (out of 88) may indicate partial PTSS, PTSS, or very high levels of PTSS [13]. Importantly, while the utility of IES-r in diagnosing posttraumatic stress disorder remains controversial especially in light of changes in its diagnostic definition, it has been validated in assessing PTSS among cancer patients [14–17].

Finally, we asked participants three questions, developed specifically for this study, to assess the impact of time on their symptoms. First, “Have you experienced any anxiety related to your surveillance scan and follow-up appointment?” with response choices consisting of not at all (0), some (1), a lot (2), or a significant amount (3). Next, participants were asked to rate their level of anxiety within the 7 days prior to their scan and then in the time period since their scan before meeting with a provider using a Likert scale (range 1–10) with higher scores representing more anxiety. Data on demographics (age, gender, race, ethnicity, marital status, education, employment, insurance status), previous mental health diagnoses, clinical details of cancer treatment, and time since diagnosis, surgery, and last scan were self-reported at the time of enrollment.

### Interview guide and process

To measure elements of scanxiety not captured by these instruments, semi-structured interviews were conducted using a convenience sample of patients. After completing the surveys, consecutive participants were invited to undergo semi-structured interviews with trained research staff to further characterize scan-associated anxiety. The interview was scheduled at a time convenient for participants after their medical visit. The interview script was developed using preliminary survey results, evidence synthesis, and expert opinion. Questions focused on the experiences of patients before, during, and after the completion of surveillance labs and imaging. Questions were open-ended and allowed for additional questions depending on the responses of the interviewees. Interviews were conducted over the phone by a research assistant with training and experience in qualitative research; they were audio recorded and then manually transcribed.

### Data analysis

Demographic and clinical characteristics were summarized using descriptive statistics. Mean and standard deviations (SD) or median and interquartile range were used to describe continuous variables and frequencies and proportions for categorical variables. Standardized scores for surveillance-associated anxiety were calculated from the responses of the HADS-anxiety and IES-r surveys and scores were summarized by sub-scale using descriptive statistics and presented as means, standard deviations, medians, and ranges [11, 13]. Higher scores represent greater anxiety and symptoms, respectively. Likert plots were used to visually summarize responses from both surveys and general questions regarding surveillance anxiety at various time points. Multivariable regression analyses were performed to identify the relationship between clinical factors and each of the studied endpoints. Possible associated variables included age, gender, race, marital status, education, distance traveled, cancer type, surgery type, metastasis, neoadjuvant/adjuvant chemotherapy, type of scan, time elapsed since surgery, time elapsed since diagnosis, scan results viewed before appointment, patient accompanied by someone at appointment, mental health diagnosis, and mental health medications taken by patient. Given the many candidate variables we identified, we used univariate analysis (variables with p > 0.1) and stepwise variable selection methods together to choose the best possible covariates for the final multivariable analysis; those with p < 0.05 were included in the final model. In the case of similar variables (i.e. mental health diagnosis and taking a mental health medication), the statistical significance and clinical relevance were compared among the variables of interest to determine which variable to include in order to mitigate collinearity. All analyses and graphs were generated in R version 4.1 and Microsoft PowerPoint.

Qualitative analysis included both an inductive and deductive coding methodology as previously described [18]. Two independent researchers read all interview transcripts as they took place and developed an initial framework using preliminary codes before a more in-depth, inductive coding process took place. This process continued iteratively until no new codes were added at which time no further interviews were conducted. Afterwards, all interviews were coded for sub-themes and the codebook was updated. These qualitative results were then integrated with the quantitative results to elucidate a more complete characterization of surveillance-associated anxiety. The study was approved by the Cancer Scientific Review Committee and Institutional Review Board.

## Results

### Patient characteristics

Among 101 prospectively enrolled patients, the mean age was 62 ± 13.9 years old and 52% were male. Most patients were White (92%) and married (76%). The most common cancer diagnoses included pancreas (24%), appendiceal (21%), and colon & rectum (21%). Mean time since surgery was 30.7 ± 28.0 months. Only 13% of patients had a self-reported mental health diagnosis while 23% were currently taking a mental health medication (Table 1). Complete data regarding patient demographics, insurance status, cancer type, surgery type, use of neoadjuvant/adjuvant therapy, prior mental health diagnosis, and details regarding surveillance visits are reported in Table 1.

**Table 1:** Demographic, clinical, and surveillance characteristics of participants

Characteristic	*N* = 101 (%)
Age, *mean (SD, range)*	61.9 (*13.9*, 27–87)
Gender	
- Male	52 (51.5)
- Female	49 (48.5)
Race	
- White	93 (92.1)
- Black	5 (5.0)
- Other	3 (3)
Ethnicity	
- Non-Hispanic	98 (97)
- Hispanic	2 (2)
- Missing	1 (1)
Marital status	
- Married	77 (76.2)
- Single	12 (11.9)
- Divorced	6 (5.9)
- Widowed	6 (5.9)
Education	
- More than high school	58 (57.4)
- Less than or equivalent to high school	42 (41.6)
- Missing	1 (1)
Employment	
- Employed	50 (49.5)
- Retired	47 (46.5)
- Unemployed	4 (4)
Insurance	
- Private	44 (43.6)
- Public	43 (42.6)
- More than one insurance	10 (9.9)
- Uninsured/missing	4 (4.0)
Cancer type	
- Pancreas	24 (23.8)
- Appendiceal	21 (20.8)
- Colorectal	21 (20.8)
- Liver/bile duct	12 (11.9)
- Esophagus/gastric	10 (9.9)
- Other	13 (13.9)
Surgery type	
- Pancreatectomy	27 (26.7)
- Hepatectomy	19 (18.8)
- Gastrectomy	10 (9.9)
- Other	45 (44.6)
Neoadjuvant therapy	
- No	74 (73.3)
- Yes	27 (26.7)
Adjuvant therapy	
- No	74 (73.3)
- Yes	27 (26.7)
Distance traveled (miles), *Mean (SD)*	54.6 (49.4)
Time (months) since diagnosis, *Mean (SD)*	34.5 (27.5)
Time (months) since surgery, *Mean (SD)*	30.7 (28.0)
Time (days) since scan, *Mean (SD)*	2.45 (5.42)
Metastasis	
- No	80 (79.2)
- Yes	21 (20.8)
Mental health diagnosis	
- No	88 (87.1)
- Yes	13 (12.9)
Taking mental health medication	
- No	78 (77.2)
- Yes	23 (22.8)
Scan results viewed before appointment	
- No	70 (69.3)
- Yes	31 (30.7)
Surveillance type	
- Imaging + Labs	57 (56.4)
- Imaging only	39 (38.6)
- Labs only	5 (5.0)
Accompanied by someone at appointment	
- Yes	56 (55.4)
- No	42 (41.6)
- Missing	3 (3.0)

### Quantitative results

**Fig. 1:**
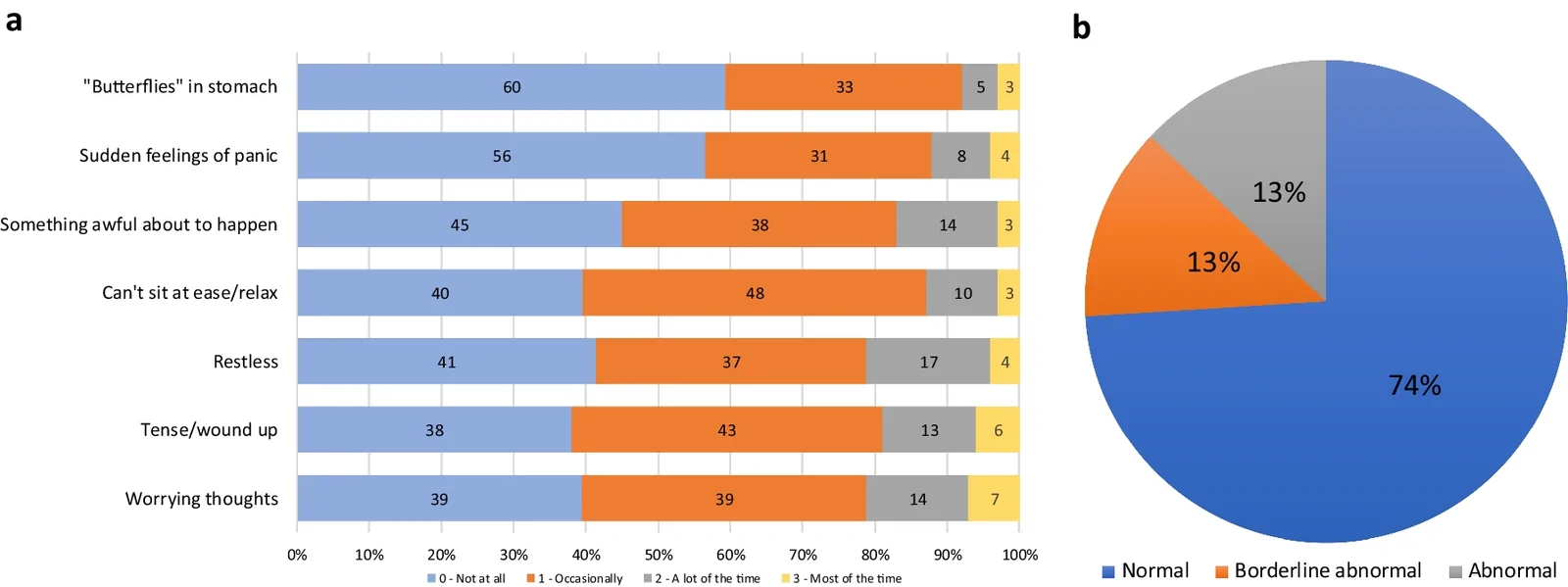
Hospital Anxiety and Depression Scale (HADS) results and summary of HADS scores

Among all patients, the mean HADS-anxiety score was 5.2 ± 4.7. Figure 1a demonstrates individual item responses ranked in order of most distressing to least distressing. The highest levels of experienced anxiety were feeling tense/wound up (62% experience at least occasionally) and having worrying thoughts (60.6% experience at least occasionally). “Butterflies” in stomach (59.4% never experienced) and sudden feelings of panic (56.6% never experienced) were the least commonly experienced by patients. Mean HADS-anxiety scores for 74% of patients were within the normal range whereas 26% of patients demonstrated borderline or abnormal levels of anxiety (Fig. 1B and Supplementary Table 1).

**Fig. 2:**
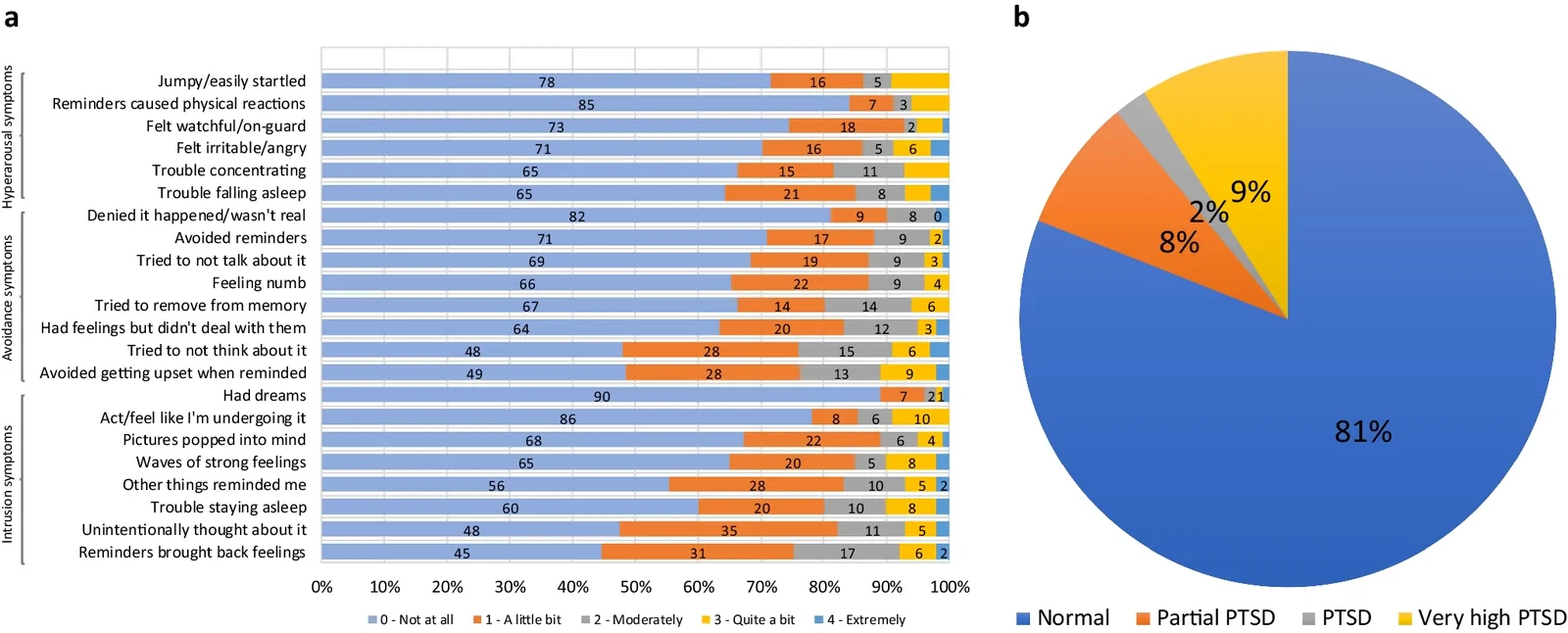
Impact of Events Scale (IES) results and Post Trauma Stress Symptoms (PTSS) severity based on IES scores

Figure 2a shows the IES-r survey results and is divided into three subscales: symptoms of hyperarousal, avoidance, and intrusion. The mean IES-r score for all patients was 12.1 ± 14.7. Symptoms most experienced by patients were reminders of bringing back previous feelings (55.4% experienced at least a little bit), avoiding getting upset when reminded (51.5% experienced at least a little bit), and trying to not think about it (52% experienced at least a little bit). While most patients (82%) had normal levels of IES-r, 18% of patients demonstrated clinically relevant levels of PTSS (Fig. 2B and Supplementary Tables 2–5).

Using the a priori established clinically relevant thresholds, 15.8% of patients had both elevated HADS-anxiety and IES-r scores; 12.9% of patients had either elevated HADS-anxiety or IES-r scores but not both; and 71.3% of patients did not have abnormal levels of either scale. Overall, 28.7% of patients experienced abnormal levels of anxiety, PTSS, or both.

**Fig. 3:**
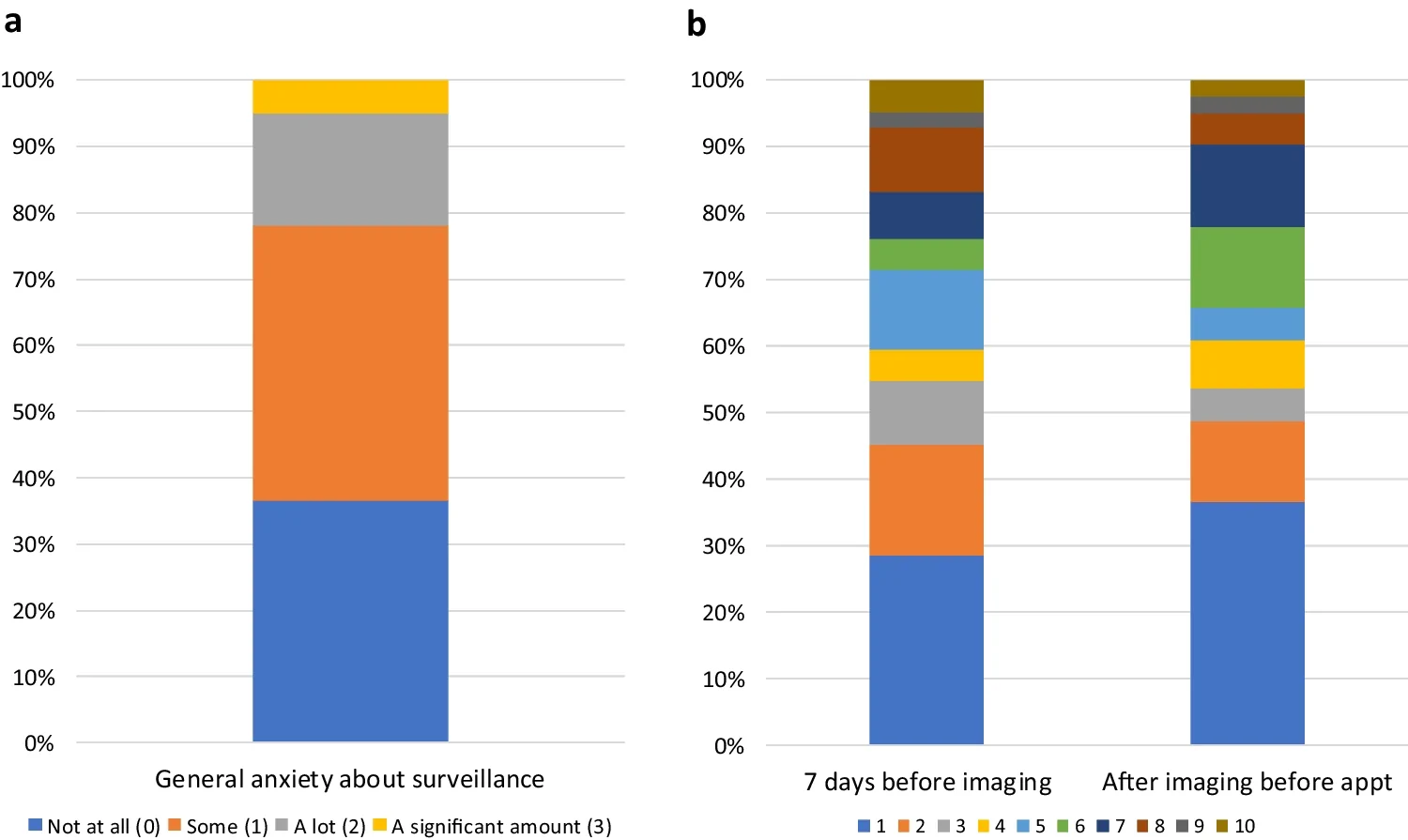
Responses to supplemental anxiety questions

In response to the question “Have you experienced any anxiety related to your surveillance scan and follow-up appointment?”, 22.0% reported “a lot” or “a significant amount” (Fig. 3A, mean score 0.9 ± 0.9). When asked about their anxiety within the 7 days prior to their scan and then in the time period since their scan before meeting with a provider, participants reported a mean 3.9 ± 2.9 (median 3, range 1–10) and mean 3.7 ± 2.8 (median 3, range 1–10, respectively (Fig. 3B) (Supplementary Table 6).

### Factors associated with HADS-anxiety and IES scores

On multivariable linear regression analysis, mental health diagnosis was associated with increased total HADS-anxiety score (β parameter = 4.3, p = 0.0008) whereas resected metastatic disease (β = −2.5, p = 0.018) and older age (years) (β = −0.1, p = 0.0023) were associated with decreased total HADS-anxiety score. When evaluating associated variables of distress measured by the IES-r, older age (years) (β = −0.5, p < 0.0001) and hepatectomy (β = −8.1, p = 0.0435) were associated with decreased total IES score whereas mental health diagnosis (β = 12.8, p = 0.0017) and public insurance (β = 10.2, p = 0.0042) were associated with increased total IES score. Results of univariate linear regression are found in Supplementary Table 7.

Multivariable linear regression was used to evaluate factors associated with IES subscales. Older age (years) was associated with decreased IES intrusion scores (β = −0.1, p = 0.0147) whereas mental health diagnosis was associated with increased IES intrusion scores (β = 5.9, p = 0.0003). Similarly, older age was associated with decreased IES avoidance scores (β = −0.1, p = 0.0064) whereas use of mental health medication was associated with increased IES avoidance scores (β = 2.5, p = 0.0484). Finally, mental health diagnosis (β = 4.5, p < 0.0001), African American race (β = 3.97, p = 0.0206), public insurance (β = 2.6, p = 0.00725), and longer distance traveled (miles) (β = 0.01, p = 0.0129) were associated with increased IES hyperarousal scores whereas older age was associated with decreased IES hyperarousal scores (β = −0.1, p < 0.0001). Results of univariate linear regression are found in Supplementary Tables 8–11.

### Qualitative results

Qualitative analysis of patient interviews identified common major themes of psychological symptoms, impact on daily living, and the importance of coping mechanisms during the surveillance period. Representative quotes for each theme are highlighted in Table 2.

**Table 2:** Qualitative results regarding scanxiety

Theme	Representative Quote
*Psychological and Physical Symptoms*	
Anxiety/Fear	"I do feel some, some nervousness as the scan approaches. Perhaps, when I get a reminder about my appointment, about 10 days before. Then I start thinking about it a little bit and then as it as it gets closer, then I do feel a little bit more anxious about it because it's important
Uncertainty	"I'm kind of like used to having the MRI or the blood work done. It's what are the results going to show and are they gonna to show that something's wrong and it preoccupies my mind a whole lot then if I keep thinking about it enough then I will like work myself up to where I like, I really get. Really freaked out about it."
Reassurance	"Once I heard Doctor [REDACTED] say that he thought everything looked great. I was very relieved. Yeah, I thought I felt like a pretty big weight has been lifted off my shoulders yesterday after the initial results of the scan."
Positive Attitude; Confidence	"I felt a certain level of confidence, everything was going to be fine. So, I was looking forward to the scan, you know, more than being discouraged by but looking forward to this scan cause I, I was looking forward to getting some results."
Negative Mood Impact	"I don't like it. I don't like the person I am at that time. It's just- it’s just not me."
Physical discomfort during scan	“The contrast dye application it, it reminds me for some reason […] of the chemotherapy […] I know it’s a big difference, but it, it just reminds me of the same thing and it makes me a little nervous because of that.”
*Impact on Social and Life Factors*	
Caregivers and loved ones	“…my wife is concerned, you know, just the same as I am […] what if it’s a bad scan, and what if they find something?”
Planning	“[I have] made more of a point to make sure we could do family vacation this summer because I, I have become more aware of my mortality and like you know what? I want to enjoy the family while I can…”
Daily Life	“Well, I guess it affects my sleep. Just in my daily routine, I think a lot about what’s coming up and wanting to get it over with and yeah, that’s kind of the feeling I have.”
*Coping and Symptom Management*	
Support from loved ones	"So then I'll send [scan results] to [my sister]. She'll be like, yeah, I agree with you. So now there's two of us and we're both like, OK, it seems to me like we're stable. I relax a little bit because at least I know the odds are better what we're both seeing it that way and I have some direction in where things stand until I talk to the doctor versus if I didn't have it at all."
New normal	“…But right now, I'm cancer free. But I know that there's a likelihood (of recurrence). So, you know, I do think about that, but I don't dwell on it, and I just go about my everyday life…"
Staying busy	“So, I just try to put on a front and try to keep myself busy and occupied to keep my mind, active and off of thinking about it."
Stress reduction strategies	"I do generally I, I, I exercise a lot at home and I, I get a nice walk every day. I think that probably helps in, in many ways with my health and perhaps also to help keep me calm during the periods when the scan is approaching […] It really does help when I, I get a good night of sleep. During this period before this the scan, it does seem to help and when I when I'm not able to, to rest very well it really affects my mood, and I think I, I, I do feel more nervous."
Trust and support from medical team	"I think there was a certain trust and confidence in [the physician's] feedback that made me think you know what he did a good job…If he says it's going to be okay, it's going to be okay."
Positive Mindset	" You know negative breeds negative you gotta you gotta you know you gotta combat it with some positive in there."
Pharmacologic	“So, I have Lexapro to help me cope, and then also I do Trazodone to help me sleep at night."
Medical Experience	"And the other thing that I think makes my case fairly unique is, I had a automobile accident back in 1989 where they gave me maybe a 5 to 10% chance of living […] I was going into this scan and this experience feeling as though if it's, if it's crappy news, if it's a bad outcome or something like that, we’re just taking this one on head on. […] when you're sort of that close to that you have a near death experience like that you, you, you walk into these things with a different kind of bravery maybe? Or a different kind of courage?"
Empathy	"Because I realized that well she’s my hero I love doctor [PHYSICIAN] she’s absolutely an amazing woman and I just adore her. And waiting two hours or three hours or four hours it doesn’t bother me because I know she’s probably with another patient holding their hand and telling them not so good news."

Psychological symptoms expressed by patients included anxiety and/or FCR during the time leading up to their scans. The onset of anxiety varied among patients, ranging from a few weeks to 24 h before the scan. One patient reported, “I do feel some, some nervousness as the scan approaches. Perhaps, when I get a reminder about my appointment, about 10 days before…..and then as it as it gets closer, then I do feel a little bit more anxious about it because it's important.” For some, this anxiety subsided immediately after the scan was finished, but for others, it went away only after receiving their test results from their provider. Several patients reported physical pain and discomfort arising from the contrast dye during the scan while others associated this sensation to receiving chemotherapy. However, not all experiences related to surveillance were negative. Some patients viewed the results of scans as a general confirmation that their treatment was effective and they remained cancer-free.

Most patients reported that their caregivers and loved ones felt anxious and uncertain about the scan outcome. Patients also explained that scanxiety impacted aspects of their daily life including sleep patterns, concentration level, and intrusive thoughts about the future. As one patient reported, “…it affects my sleep. Just in my daily routine, I think a lot about what’s coming up and wanting to get it over with…” Additionally, some patients expressed that traveling to appointments was an added burden when they lived far from the medical center. As a result of these experiences, most patients preferred to minimize the time between their scan and discussing results with a provider.

Patients utilized a range of coping strategies to manage potential scanxiety with support from loved ones being most common especially while waiting for the results. Surveillance-associated anxiety was accepted as the new normal and an unavoidable aspect of survivorship. One patient mentioned, “…But right now, I'm cancer free. But I know that there's a likelihood [of recurrence]. So, you know, I do think about that, but I don't dwell on it, and I just go about my everyday life…" Other coping strategies included staying busy and employing stress reduction strategies. More than one-third of patients reported trust in their oncologist’s surveillance approach and appreciated their clinical team’s efforts to make them comfortable during the scan procedure. In contrast, several found having a positive outlook helped their ability to cope with anxiety. Some relied on their previous medical experience, either as medical professionals or as patients to ease their uncertainty surrounding scan results. Lastly, a few reported pharmacologic use, and two participants displayed empathy toward others going through cancer treatments.

## Discussion

In this cross-sectional mixed-methods study of patients with resected GI and HPB cancers using validated measures of anxiety and post-traumatic stress symptoms, scanxiety was common but relatively mild during routine cancer surveillance. Yet, more than 25% of patients in our study experienced clinically relevant levels of anxiety and/or PTSS. A prior mental health diagnosis and other pertinent clinical factors were associated with worse symptoms, suggesting the potential for targeted interventions in high-risk patients. Given the increasing number of cancer survivors, additional research is needed on patient-centered strategies to prevent and reduce scanxiety and improve quality of life among individuals undergoing cancer-related surveillance.

Postoperative surveillance plays a critical role in the ongoing care of cancer survivors. The purpose of frequent surveillance is early detection of recurrence; however, the process of undergoing imaging, labs, and follow-up visits may be distressing to patients because of the high stakes of the results [19]. In turn, upcoming imaging and labs can cause varying degrees of psychological symptoms including anxiety, depression, and FCR [5, 20]. Although scanxiety has been extensively studied in cancer patients receiving ongoing treatment, few studies to date have examined this phenomenon in patients after curative intent GI and HPB surgery. Understanding scanxiety in this patient population is critical since these patients have elevated risks of recurrence and cancer-related mortality but also the surveillance period typically lasts for many years after surgery and can have a significant impact on overall quality of life [21, 22].

In this study, we investigated the severity and timing of symptoms in relation to imaging and sought to identify patient factors associated with increased scanxiety. We found that anxiety was common among patients (63.4% of patients on direct elicitation reported some anxiety surrounding surveillance) and yet overall symptoms were mild (mean HADS-anxiety scores low). The prevalence of any degree of anxiety in our study is similar to reported studies in breast and gynecologic oncology literature in which 34% to 85% of study participants experienced surveillance-associated anxiety [23–26]. Additionally, in our semi-structured interviews, we found that patient anxiety was most severe during the period immediately before the scan until after the scheduled follow-up visit, which is consistent with previous longitudinal studies [20, 27–29]. The duration of scanxiety varied as some patients reported anxiety beginning weeks to 24 h before imaging and lasted anywhere from immediately after the scan was completed to after reviewing results with a physician.

The range of patient experiences observed is also consistent with previously reported studies. For example, previous literature in urologic, lung, and esophageal cancers have reported the unique nature of a patient’s experience during surveillance and that this anxiety was commonly linked to uncertainty about results and subsequent FCR [3, 8, 21, 30, 31]. In our study, patients frequently mentioned coping strategies such as staying busy, relying on family/friends as a support system, and viewing imaging as a “new normal” with the goal of these coping mechanisms to reduce overall scanxiety. Most patients preferred to have short intervals between scans and office visits because there was less opportunity for confusion or misinterpretation of results. Consequently, patients recommended decreasing the scan-to-results waiting period or updating patients on expected clinic visit wait times as interventions to reduce overall scanxiety.

Although anxiety scores were low overall among patients in this study, we identified several patient factors that were consistently associated with increased levels of anxiety and PTS including previous mental health diagnosis and younger age. We found that social determinants of health factors including race, insurance status, and travel distance were associated with increased hyperarousal scores, which further supports the need for intervention in these populations [32]. On the other hand, several factors including gender, marital status, education, cancer type, surveillance type, and others were not found to be associated with increasing symptoms. Indeed, the observation that cancer type was not associated suggests that this research may be broadly generalizable. Future studies should focus on screening for patients at risk for increased scanxiety with the goal of early intervention during surveillance. Interventions may include early participation in survivor support groups or designating a clinic liaison to help guide patients through survivorship. Additionally, at-risk patients may benefit from psychosocial support to help improve coping mechanisms with the goal of better anxiety management [33].

Although the above findings are noteworthy, several limitations should be acknowledged. Our cohort included patients with similar demographics who were treated at a single tertiary cancer center, and as a result, our findings may not be generalizable to other, diverse populations. For instance, we used a small sample to conduct semi-structured interviews which limits the generalizability of these findings. Additionally, this cross-sectional analysis describes patient experiences at a single point in time and does not characterize changes in scanxiety that may occur over time. In particular, some questions asked patients to recall their anxiety as a previous time point which may be especially prone to bias. Furthermore, while our findings are strengthened by the inclusion of qualitative analyses, some multidimensional aspects of scanxiety may be missed due to the inclusion of only the HADS-anxiety and IES-r scales.

In conclusion, scanxiety among patients undergoing surveillance for resected GI and HPB cancers was common but relatively mild although approximately 25% experienced clinically significant symptoms. Future research should focus on developing interventions for patients at highest risk of surveillance-associated anxiety with the goal of reducing distress and improving overall quality of life during cancer survivorship.

## Supplementary Information

Below is the link to the electronic supplementary material.

Supplementary file1 (DOCX 57 KB)

## Data Availability

No datasets were generated or analysed during the current study.
