# Supplementary material for: Characterizing surveillance-associated anxiety following curative-intent surgery for gastrointestinal cancer: a mixed methods approach
Source: J Cancer Surviv. 2025 Apr 29;20(Suppl 1):229–38. doi: 10.1007/s11764-025-01781-w (PMC13676524; doi:10.1007/s11764-025-01781-w)
Supplement: Supplementary file 1 [file 11764_2025_1781_MOESM1_ESM.docx]

**Supplementary Tables**

**Supplementary Table 1. Summary of HADS Scores**

|  | **Butterflies in stomach** | **Sudden feelings of panic** | **Something awful about to happen** | **Can’t sit at ease/relax** | **Restless** | **Tense/wound up** | **Worrying thoughts** | **Total** |
| --- | --- | --- | --- | --- | --- | --- | --- | --- |
| **Mean ± SD** | 0.5 ± 0.7 | 0.6 ± 0.8 | 0.8 ± 0.8 | 0.8 ± 0.8 | 0.8 ± 0.9 | 0.9 ± 0.9 | 0.9 ± 0.9 | 5.2 ± 4.7 |
| **Median** | 0 | 0 | 1 | 1 | 1 | 1 | 1 | 4 |
| **Range** | 0-3 | 0-3 | 0-3 | 0-3 | 0-3 | 0-3 | 0-3 | 0-21 |
| **Frequency, N (%)**   - **0** - **1** - **2** - **3** | 60 (59.4)  33 (32.7)  5 (4.9)  3 (3.0) | 56 (56.6)  31 (31.3)  8 (8.1)  4 (4.0) | 45 (45.0)  38 (38.0)  14 (14.0)  3 (3.0) | 40 (39.6)  48 (47.5)  10 (9.9)  3 (3.0) | 41 (41.4)  37 (37.4)  17 (17.2)  4 (4.0) | 38 (38.0)  43 (43.0)  13 (13.0)  6 (6.0) | 39 (39.4)  39 (39.4)  14 (14.1)  7 (7.1) |  |
| **Total HADS-anxiety score, N (%)**  - Normal (0-7)  - Borderline abnormal (8-10)  - Abnormal (11-21) |  |  |  |  |  |  |  | 75 (74.3)  13 (12.9  13 (12.9) |

**Supplementary Table 2. Summary of Impact of Events Scale Total Scores**

|  | **Total, N (%)** |
| --- | --- |
| **Total IES-r score**   - Normal (0-23) - Partial PTSS (24-32) - PTSS (33-36) - Very high PTSS (37-88) | 82 (81.2)  8 (7.9)  2 (2.0)  9 (8.9) |

**Supplementary Table 3. Summary of Impact of Events Scale (IES) Hyperarousal Scores**

| **Hyperarousal symptoms** | **Jumpy/easily startled** | **Reminders caused physical reactions** | **Felt watchful** | **Felt irritable** | **Trouble concentrating** | **Trouble falling asleep** |
| --- | --- | --- | --- | --- | --- | --- |
| **Mean ± SD** | 0.3 ± 0.6 | 0.3 ± 0.8 | 0.4 ± 0.8 | 0.6 ± 1.0 | 0.6 ± 1.0 | 0.6 ± 1.0 |
| **Median** | 0 | 0 | 0 | 0 | 0 | 0 |
| **Range** | 0-3 | 0-3 | 0-4 | 0-4 | 0-3 | 0-4 |
| **Frequency, N (%)**   - **0** - **1** - **2** - **3** - **4** | 78 (78.0)  16 (16.0)  5 (5.0)  1 (1.0)  0 (0.0) | 85 (84.2)  7 (6.9)  3 (3.0)  6 (5.9)  0 (0.0) | 73 (74.5)  18 (18.4)  2 (2.0)  4 (4.1)  1 (1.0) | 71 (70.3)  16 (15.8)  5 (5.0)  6 (5.9)  3 (3.0) | 65 (66.3)  15 (15.3)  11 (11.2)  7 (7.1)  0 (0.0) | 65 (64.4)  21 (20.8)  8 (7.9)  4 (4.0)  3 (3.0) |

**Supplementary Table 4. Summary of Impact of Events Scale (IES) Avoidance Scores**

| **Avoidance symptoms** | **Denied it happened** | **Avoided reminders** | **Tried to not talk about it** | **Feeling numb** | **Tried to remove from memory** | **Had feelings but didn’t deal with them** | **Tried to not think about it** | **Avoided getting upset when reminded** |
| --- | --- | --- | --- | --- | --- | --- | --- | --- |
| **Mean ± SD** | 0.3 ± 0.8 | 0.5 ± 0.8 | 0.5 ± 0.9 | 0.5 ± 0.8 | 0.6 ± 0.9 | 0.6 ± 0.9 | 0.9 ± 1.1 | 0.9 ± 1.1 |
| **Median** | 0 | 0 | 0 | 0 | 0 | 0 | 1 | 1 |
| **Range** | 0-4 | 0-4 | 0-4 | 0-3 | 0-3 | 0-4 | 0-4 | 0-4 |
| **Frequency, N (%)**   - **0** - **1** - **2** - **3** - **4** | 82 (81.2)  9 (8.9)  8 (7.9)  0 (0.0)  2 (2.0) | 71 (71.0)  17 (17.0)  9 (9.0)  2 (2.0)  1 (1.0) | 69 (68.3)  19 (18.8)  9 (8.9)  3 (3.0)  1 (1.0) | 66 (65.3)  22 (21.8)  9 (8.9)  4 (4.0)  0 (0.0) | 67 (66.3)  14 (13.9)  14 (13.9)  6 (5.9)  0 (0.0) | 64 (63.4)  20 (19.8)  12 (11.9)  3 (3.0)  2 (2.0) | 48 (48.0)  28 (28.0)  15 (15.0)  6 (6.0)  3 (3.0) | 49 (48.5)  28 (27.7)  13 (12.9)  9 (8.9)  2 (2.0) |

**Supplementary Table 5. Summary of Impact of Events Scale (IES) Intrusion Scores**

| **Intrusion symptoms** | **Had dreams** | **Act/feel like I’m undergoing it** | **Pictures popped into mind** | **Waves of strong feelings** | **Other things reminded me** | **Trouble staying asleep** | **Unintentionally thought about it** | **Reminders brought back feelings** |
| --- | --- | --- | --- | --- | --- | --- | --- | --- |
| **Mean ± SD** | 0.2 ± 0.6 | 0.2 ± 0.6 | 0.5 ± 0.9 | 0.6 ± 1.0 | 0.7 ± 1.0 | 0.7 ± 1.1 | 0.8 ± 1.0 | 0.9 ± 1.0 |
| **Median** | 0 | 0 | 0 | 0 | 0 | 0 | 1 | 1 |
| **Range** | 0-4 | 0-3 | 0-4 | 0-4 | 0-4 | 0-4 | 0-4 | 0-4 |
| **Frequency, N (%)**   - **0** - **1** - **2** - **3** - **4** | 90 (89.1)  7 (6.9)  2 (2.0)  1 (1.0)  1 (1.0) | 86 (85.1)  8 (7.9)  6 (5.9)  1 (1.0)  0 (0.0) | 68 (67.3)  22 (21.8)  6 (5.9)  4 (4.0)  1 (1.0) | 65 (65.0)  20 (20.0)  5 (5.0)  8 (8.0)  2 (2.0) | 56 (55.4)  28 (27.7)  10 (9.9)  5 (5.0)  2 (2.0) | 60 (60.0)  20 (20.0)  10 (10.0)  8 (8.0)  2 (2.0) | 48 (47.5)  35 (34.7)  11 (10.9)  5 (5.0)  2 (2.0) | 45 (44.6)  31 (30.7)  17 (16.8)  6 (5.9)  2 (2.0) |

**Supplementary Table 6. Summary of Responses to Supplemental Anxiety Questions**

| **N = 41** | **General anxiety about surveillance** | **Anxiety 7 days before imaging** | **Anxiety after imaging but before provider appointment** |
| --- | --- | --- | --- |
| **Mean ± SD** | 0.9 ± 0.9 | 4 ± 2.9 | 3.8 ± 2.8 |
| **Median** | 1 | 3 | 3 |
| **Range** | 0-3 | 1-10 | 1-10 |
| **Frequency, N (%)**   - **0** - **1** - **2** - **3** - **4** - **5** - **6** - **7** - **8** - **9** - **10** | 15 (36.6)  17 (41.5)  7 (17.1)  2 (4.9)  --  --  --  --  --  --  -- | --  12 (28.6)  7 (16.7)  4 (9.5)  2 (4.8)  5 (11.9)  2 (4.8)  3 (7.1)  4 (9.5)  1 (2.4)  2 (4.8) | --  15 (36.6)  5 (12.2)  2 (4.9)  3 (7.3)  2 (4.9)  5 (12.2)  5 (12.2)  2 (4.9)  1 (2.4)  1 (2.4) |

**Supplemental Table 7. Univariate Regression Analysis for HADS Total Score**

| **Characteristic** | **Univariate Regression Analysis** | |
| --- | --- | --- |
|  | Coefficient (Std.Error) | P-value |
| **Age** | -0.11 (0.03) | 0.001 |
| **Distance Traveled** | 0.007 (0.01) | 0.429 |
| **Time elapsed since surgery** | 0.0003 (0.001) | 0.557 |
| **Time elapsed since diagnosis** | 0.0003 (0.63) | 0.530 |
| **Time elapsed since scan** | 0.13 (0.09) | 0.123 |
| **Gender**   - Female - Male | Reference  -1.24 (0.92) | 0.183 |
| **Race**   - White - African American - Other | Reference  0.44 (2.16)  -0.83 (2.76) | 0.839  0.765 |
| **Ethnicity**   - Hispanic - Non-Hispanic | Reference  -2.41 (3.35) | 0.474 |
| **Education**   - Less than or equivalent to High school - More than High school | Reference  -0.14 (0.95) | 0.882 |
| **Insurance**   - Private - Public - More than one insurance - Uninsured | Reference  -0.30 (1.01)  0.17 (1.65)  1.02 (2.47) | 0.770  0.917  0.679 |
| **Employment**   - Employed - Unemployed - Retired | Reference  0.35 (2.41)  -1.62 (0.94) | 0.885  0.088 |
| **Cancer type**   - Colorectal - Esophagus/Gastric - Liver - Pancreas - Other | Reference  -2.4 (1.82)  -0.88 (1.89)  -0.77 (1.43)  -1.18 (1.30) | 0.191  0.643  0.367  0.592 |
| **Metastasis**   - Yes - No | Reference  1.94 (1.13) | 0.089 |
| **Surgery type**   - Esophagectomy/Gastrectomy - Hepatectomy - Pancreatectomy - Multiple surgeries - Other | Reference  3.63 (2.87)  -3.13 (4.97)  0.60 (1.83)  1.38 (1.78) | 0.209  0.531  0.741  0.439 |
| **Adjuvant therapy**   - No - Yes | Reference  -0.37 (1.05) | 0.727 |
| **Neoadjuvant therapy**   - No - Yes | Reference  -1.03 (1.05) | 0.330 |
| **Mental health diagnosis**   - No - Yes | Reference  5.03 (1.30) | <0.001 |
| **Taking mental health medication**   - No - Yes | Reference  4.97 (0.99) | <0.001 |
| **Scan results viewed ahead of time**   - No - Yes | Reference  2.19 (0.99) | 0.029 |
| **Scan type (Imaging analysis type)**   - Both (Imaging + Labs) - Imaging - Labs | Reference  0.06 (0.98)  -0.78 (2.19) | 0.955  0.725 |
| **Accompanied by someone at the scan**   - No - Yes | Reference  0.60 (0.95) | 0.530 |

**Supplemental Table 8. Univariate Regression Analysis for IES Total Score**

|  | **Univariate Regression Analysis** | |
| --- | --- | --- |
|  | Coefficient (Std.Error) | P-value |
| **Age** | -0.34 (0.10) | <0.001 |
| **Distance Traveled** | 0.02 (0.03) | 0.457 |
| **Time elapsed since surgery** | 0.0007 (0.0001) | 0.700 |
| **Time elapsed since diagnosis** | 0.0004 (0.002) | 0.800 |
| **Time elapsed since scan** | 0.23 (0.27) | 0.407 |
| **Gender**   - Female - Male | Reference  -1.83 (2.94) | 0.535 |
| **Race**   - White - African American - Other | Reference  9.14 (6.76)  -1.66 (8.63) | 0.179  0.848 |
| **Ethnicity**   - Hispanic - Non-Hispanic | Reference  -17.91 (10.43) | 0.089 |
| **Education**   - Less than or equivalent to High school - More than High school | Reference  -0.67 (3.00) | 0.824 |
| **Insurance**   - Private - Public - More than one insurance - Uninsured | Reference  0.32 (0.92)  -7.43 (5.17)  -1.73 (7.70) | 0.920  0.154  0.823 |
| **Employment**   - Employed - Unemployed - Retired | Reference  2.89 (7.67)  -3.04 (3.00) | 0.707  0.313 |
| **Cancer type**   - Colorectal - Esophagus/Gastric - Liver - Pancreas - Other | Reference  -6.60 (5.97)  -5.14 (5.97)  -3.28 (4.51)  -1.99 (4.11) | 0.255  0.391  0.468  0.629 |
| **Metastasis**   - Yes - No | Reference  4.46 (3.60) | 0.217 |
| **Surgery type**   - Esophagectomy/Gastrectomy - Hepatectomy - Pancreatectomy - Multiple surgeries - Other | Reference  11.13 (8.98)  -7.63 (15.55)  0.59 (5.72)  5.65 (5.57) | 0.218  0.625  0.918  0.313 |
| **Adjuvant therapy**   - No - Yes | Reference  2.45 (3.31) | 0.462 |
| **Neoadjuvant therapy**   - No - Yes | Reference  -2.86 (3.31) | 0.390 |
| **Mental health diagnosis**   - No - Yes | Reference  15.29 (4.11) | <0.001 |
| **Taking mental health medication**   - No - Yes | Reference  12.65 (3.27) | <0.001 |
| **Scan results viewed ahead of time**   - No - Yes | Reference  3.59 (3.17) | 0.260 |
| **Scan type (Imaging analysis type)**   - Both (Imaging + Labs) - Imaging - Labs | Reference  -2.47 (3.07)  -4.01 (6.90) | 0.424  0.562 |
| **Accompanied by someone at the scan**   - No - Yes | Reference  3.29 (3.02) | 0.280 |

**Supplemental Table 9. Univariate Regression Analysis for IES Intrusion Score**

|  | **Univariate Regression Analysis** | |
| --- | --- | --- |
|  | Coefficient (Std.Error) | P-value |
| **Age** | -0.12 (0.04) | 0.003 |
| **Distance Traveled** | 0.005 (0.01) | 0.671 |
| **Time elapsed since surgery** | 0.0005 (0.0007) | 0.486 |
| **Time elapsed since diagnosis** | 0.0004 (0.0007) | 0.577 |
| **Time elapsed since scan** | 0.08 (0.11) | 0.477 |
| **Gender**   - Female - Male | Reference  -0.73 (1.16) | 0.530 |
| **Race**   - White - African American - Other | Reference  4.51 (2.64)  -3.16 (3.37) | 0.091  0.351 |
| **Ethnicity**   - Hispanic - Non-Hispanic | Reference  -6.03 (4.13) | 0.148 |
| **Education**   - Less than or equivalent to high school - More than high school | Reference  -0.50 (1.18) | 0.671 |
| **Insurance**   - Private - Public - More than one insurance - Uninsured | Reference  0.34 (1.25)  -2.82 (2.04)  -1.57 (3.03) | 0.783  0.169  0.606 |
| **Employment**   - Employed - Unemployed - Retired | Reference  2.52 (3.02)  -0.98 (1.18) | 0.406  0.408 |
| **Cancer type**   - Colorectal - Esophagus/Gastric - Liver - Pancreas - Other | Reference  -3.30 (2.26)  -2.26 (2.34)  -1.63 (1.77)  -1.16 (1.61) | 0.148  0.278  0.360  0.475 |
| **Metastasis**   - Yes - No | Reference  2.29 (1.41) | 0.108 |
| **Surgery type**   - Esophagectomy/Gastrectomy - Hepatectomy - Pancreatectomy - Multiple surgeries - Other | Reference  2.63 (3.57)  -1.88 (6.19)  0.96 (2.27)  2.60 (2.22) | 0.464  0.762  0.673  0.245 |
| **Adjuvant therapy**   - No - Yes | Reference  1.12 (1.31) | 0.393 |
| **Neoadjuvant therapy**   - No - Yes | Reference  -1.56 (1.30) | 0.234 |
| **Mental health diagnosis**   - No - Yes | Reference  6.70 (1.60) | <0.001 |
| **Taking mental health medication**   - No - Yes | Reference  5.27 (1.28) | <0.001 |
| **Scan results viewed ahead of time**   - No - Yes | Reference  1.61 (1.25) | 0.199 |
| **Scan type (Imaging analysis type)**   - Both (Imaging + Labs) - Imaging - Labs | Reference  -1.22 (1.21)  -0.94 (2.72) | 0.317  0.730 |
| **Accompanied by someone at the scan**   - No - Yes | Reference  1.27 (1.19) | 0.291 |

**Supplemental Table 10. Univariate Regression Analysis for IES Avoidance Score**

|  | **Univariate Regression Analysis** | |
| --- | --- | --- |
|  | Coefficient (Std.Error) | P-value |
| **Age** | -0.12 (0.04) | 0.002 |
| **Distance Traveled** | 0.01 (0.01) | 0.308 |
| **Time elapsed since surgery** | -0.0001 (0.0007) | 0.829 |
| **Time elapsed since diagnosis** | -0.0002 (0.0007) | 0.756 |
| **Time elapsed since scan** | 0.06 (0.10) | 0.540 |
| **Gender**   - Female - Male | Reference  -0.60 (1.10) | 0.589 |
| **Race**   - White - African American - Other | Reference  0.75 (2.56)  1.69 (3.27) | 0.769  0.607 |
| **Ethnicity**   - Hispanic - Non-Hispanic | Reference  -4.92 (3.94) | 0.215 |
| **Education**   - Less than or equivalent to high school - More than high school | Reference  0.42 (1.13) | 0.707 |
| **Insurance**   - Private - Public - More than one insurance - Uninsured | Reference  -0.51 (1.19)  -3.25 (1.94)  0.50 (2.89) | 0.671  0.100  0.863 |
| **Employment**   - Employed - Unemployed - Retired | Reference  -0.98 (2.87)  -1.52 (1.12) | 0.734  0.179 |
| **Cancer type**   - Colorectal - Esophagus/Gastric - Liver - Pancreas - Other | Reference  -0.85 (2.18)  -0.33 (2.25)  0.12 (1.70)  0.71 (1.55) | 0.697  0.885  0.945  0.647 |
| **Metastasis**   - Yes - No | Reference  0.68 (1.36) | 0.616 |
| **Surgery type**   - Esophagectomy/Gastrectomy - Hepatectomy - Pancreatectomy - Multiple surgeries - Other | Reference  5.13 (3.36)  -3.63 (5.82)  0.08 (2.14)  1.81 (2.09) | 0.131  0.535  0.971  0.389 |
| **Adjuvant therapy**   - No - Yes | Reference  0.37 (1.25) | 0.771 |
| **Neoadjuvant therapy**   - No - Yes | Reference  -0.19 (1.25) | 0.879 |
| **Mental health diagnosis**   - No - Yes | Reference  3.22 (1.62) | 0.049 |
| **Taking mental health medication**   - No - Yes | Reference  3.33 (1.27) | 0.010 |
| **Scan results viewed ahead of time**   - No - Yes | Reference  1.22 (1.19) | 0.307 |
| **Scan type (Imaging analysis type)**   - Both (Imaging + Labs) - Imaging - Labs | Reference  -0.38 (1.15)  -2.40 (2.59) | 0.740  0.357 |
| **Accompanied by someone at the scan**   - No - Yes | Reference  1.04 (1.14) | 0.365 |

**Supplemental Table 11. Univariate Regression Analysis for IES Hyperarousal Score**

|  | **Univariate Regression Analysis** | |
| --- | --- | --- |
|  | Coefficient (Std.Error) | P-value |
| **Age** | -0.10 (0.03) | 0.002 |
| **Distance Traveled** | 0.006 (0.009) | 0.506 |
| **Time elapsed since surgery** | 0.0003 (0.0005) | 0.512 |
| **Time elapsed since diagnosis** | 0.0003 (0.0005) | 0.607 |
| **Time elapsed since scan** | 0.09 (0.80) | 0.275 |
| **Gender**   - Female - Male | Reference  -0.50 (0.86) | 0.563 |
| **Race**   - White - African American - Other | Reference  3.88 (1.95)  -0.18 (2.50) | 0.0495  0.942 |
| **Ethnicity**   - Hispanic - Non-Hispanic | Reference  -6.96 (3.01) | 0.023 |
| **Education**   - Less than or equivalent to high school - More than high school | Reference  -0.59 (0.88) | 0.503 |
| **Insurance**   - Private - Public - More than one insurance - Uninsured | Reference  0.48 (0.93)  -1.36 (1.52)  -0.66 (2.26) | 0.606  0.372  0.771 |
| **Employment**   - Employed - Unemployed - Retired | Reference  1.35 (2.24)  -0.54 (0.88) | 0.549  0.541 |
| **Cancer type**   - Colorectal - Esophagus/Gastric - Liver - Pancreas - Other | Reference  -2.54 (1.67)  -2.26 (1.73)  -1.78 (1.31)  -1.55 (1.19) | 0.146  0.194  0.177  0.198 |
| **Metastasis**   - Yes - No | Reference  1.49 (1.05) | 0.159 |
| **Surgery type**   - Esophagectomy/Gastrectomy - Hepatectomy - Pancreatectomy - Multiple surgeries - Other | Reference  3.38 (2.61)  -2.13 (4.52)  -0.45 (1.66)  1.25 (1.62) | 0.199  0.639  0.787  0.443 |
| **Adjuvant therapy**   - No - Yes | Reference  0.96 (0.97) | 0.322 |
| **Neoadjuvant therapy**   - No - Yes | Reference  -1.11 (0.96) | 0.252 |
| **Mental health diagnosis**   - No - Yes | Reference  5.37 (1.16) | <0.001 |
| **Taking mental health medication**   - No - Yes | Reference  4.04 (0.94) | <0.001 |
| **Scan results viewed ahead of time**   - No - Yes | Reference  0.75 (0.93) | 0.418 |
| **Scan type (Imaging analysis type)**   - Both (Imaging + Labs) - Imaging - Labs | Reference  -0.87 (0.90)  -0.67 (2.01) | 0.337  0.740 |
| **Accompanied by someone at the scan**   - No - Yes | Reference  0.98 (0.88) | 0.270 |
